# Editorial: Extracorporeal cardio-pulmonary resuscitation (ECPR)

**DOI:** 10.3389/fcvm.2024.1528485

**Published:** 2024-11-26

**Authors:** Tharusan Thevathasan, Gary Schwartz, Helle Søholm

**Affiliations:** ^1^ Department of Cardiology, Angiology and Intensive Care Medicine, Deutsches Herzzentrum der Charité (DHZC), Campus Benjamin Franklin, Berlin, Germany; ^2^ Berlin Institute of Health (BIH), Charité - Universitätsmedizin Berlin, Berlin, Germany; ^3^ DZHK (German Centre for Cardiovascular Research), Partner Site Berlin, Berlin, Germany; ^4^ Department of Thoracic Surgery, Baylor University Medical Center, Dallas, TX, United States; ^5^ Department of Cardiology, The Heart Centre, Copenhagen University Hospital - Rigshospitalet, Copenhagen, Denmark; ^6^ Department of Cardiology, Skånes University Hospital, Lund, Sweden

**Keywords:** cardiac arrest, extracorporeal cardiopulmonary resuscitation, veno-arterial extracorporeal membrane oxygenation, acute cardiac care, resuscitation

**Editorial on the Research Topic**
Extracorporeal cardio-pulmonary resuscitation (ECPR)

Extracorporeal cardiopulmonary resuscitation (ECPR) has emerged as a potentially effective treatment strategy in the management of refractory cardiac arrest in selected patients, complementing conventional cardiopulmonary resuscitation (CPR). Its strategic implementation aims to stabilize hemodynamics while underlying causes are addressed, as a last resort treatment option. Recent studies highlight developments, challenges and future directions in the field of ECPR.

A cornerstone of current ECPR research lies in identifying factors that predict patient outcomes and refining decision-making tools for its initiation as the treatment modality comes with a cost, both as potential patient harm and financially. For instance, a study conducted at the Montreal Heart Institute in Canada evaluated a decision-making algorithm based on clinical variables, such as age, arrest rhythm, “no-flow” time and serum lactate levels, highlighting that strict adherence to these criteria correlated with potentially improved survival rates and favorable neurological outcomes - though in a relatively small cohort. Gradual deviations from these criteria were associated with diminished outcomes, underscoring the importance of strict patient selection to enhance ECPR efficacy. Notably, similar findings were echoed in trials, such as ARREST, Prague-OHCA and INCEPTION, which have demonstrated contrasting yet complementary insights into patient selection, procedural timing and the potential for ECPR's efficacy relative to conventional advanced cardiac life support (ACLS) (1–3).

Complementing these efforts, advancements in predictive methodologies, particularly with the integration of machine learning (ML), have made significant strides. A single-center study from the Samsung Medical Center in Korea demonstrated the power of ML by employing multiple algorithms to predict neurological outcomes in ECPR-treated patients, with the *eXtreme Gradient Boosting* model proving most effective. This approach identified mean blood pressure, initial serum lactate and time until ECPR as key predictors, showcasing ML's potential to tailor ECPR strategies and emphasizing the need for data-driven tools that potentially optimize patient care and resource allocation. However, it is essential to recognize that these tools should be readily accessible and serve as a complement to clinical practice, rather than causing any delay in its execution.

Noteworthy, the integration of ECPR eligibility tools and ML innovations must be contextualized within patient-specific factors, such as sex, that might further influence ECPR success. A single-center study from Hamburg in Germany on sex-based differences revealed that female patients undergoing ECPR for out-of-hospital cardiac arrest (OHCA) demonstrated higher survival rates with favorable neurological outcomes compared to males. This disparity was presumably linked to higher rates of witnessed cardiac events and bystander CPR in women, emphasizing that public health measures and awareness campaigns could potentially play an essential role in addressing these differences and ensuring equitable care. Additionally, procedural nuances, such as the method of CPR administered before ECPR, might further influence outcomes. Research from the same medical center investigating the impact of mechanical CPR prior to ECPR revealed it may not consistently enhance survival and could be associated with delays that prolong pre-ECMO times, underscoring the importance of streamlining pre-ECPR management. All in all, patient demographics, procedural elements and pre-cannulation interventions might significantly impact outcomes. For example, findings from the INCEPTION trial highlighted challenges in multicenter implementation, such as variability in cannulation expertise and prolonged “low-flow” times, which may have correlated with suboptimal survival rates compared to single-center studies like ARREST. This suggests that centralization of ECPR expertise and targeted ECPR training programs across multiple sites may be necessary to achieve consistent results.

The discussion of ECPR's applicability extends beyond standard resuscitative efforts to specialized contexts such as high-risk percutaneous coronary intervention (PCI). Findings from a small single-center study at the Zhengzhou University in China underscored the potential life-saving impact of standby ECMO in reducing “low-flow” time and associated complications during high-risk procedures. These findings are further substantiated by evidence from other institutions (4). Such studies suggest proactive ECPR protocols in defined scenarios, supported by well-established systems of care capable of timely patient transfer and rapid intervention.

The evaluation of hemodynamic parameters also plays a critical role in guiding ECPR management and decisions regarding decannulation. A recent case series from Minnesota in the United States explored the correlation between pulmonary capillary wedge pressure (PCWP) and left-ventricular end-diastolic pressure (LVEDP) in patients on VA-ECMO. The findings highlighted that PCWP may not reliably reflect LVEDP, particularly at higher ECMO flow rates. These findings may call for a dual approach in monitoring hemodynamic parameters to inform the management of VA-ECMO and potential unloading strategies, ensuring nuanced patient care.

This research topic is concluded by two compelling cases that illustrate the life-saving potential and adaptability of ECPR in diverse clinical emergencies: Fulminant myocarditis (FM) exemplifies the urgent need for rapid and effective interventions due to its acute onset and potentially severe hemodyamic consequences, which might lead to cardiac arrest with a high mortality rate (5). A recent case highlighted the potential of ECPR in a young adult with FM who underwent 120 min of mechanical CPR prior to the initiation of VA-ECMO, ultimately achieving complete recovery without neurological deficits. However, it is important to emphasize that the time to ECMO initiation should be minimized to optimize survival probabilities. This case represents an outlier, illustrating an exceptional rather than typical outcome. Another illustrative case detailed the use of ECPR in a young patient experiencing refractory cardiogenic shock/arrest induced by aluminum phosphide poisoning. Despite severe complications, including acute respiratory distress syndrome and sepsis, the integration of VA-ECMO with supportive therapies led to a complete recovery. These cases underscore ECPR's potential capacity to manage a range of critical scenarios, reinforcing its adaptability in treating complex clinical emergencies. They highlight the importance of timely intervention, coordinated teamwork and strategic use of mechanical circulatory support.

These recent research findings elucidate several critical points. Firstly, patient selection remains a fundamental determinant of ECPR success, potentially supported by clinical decision-making algorithms and innovations, such as ML, that may enhance predictive accuracy. Secondly, demographic and procedural insights, such as sex disparities and tailored strategies for high-risk procedures like PCI, emphasize the necessity of personalized care approaches across different spectrums. Thirdly, hemodynamic monitoring in VA-ECMO patients, including challenges such as the discrepancy between PCWP and LVEDP, necessitates ongoing research and the refinement of assessment protocols. ECPR continues to be a frontier of hope and complexity in resuscitating patients during refractory cardiac arrest. Its successful implementation may rely on the integration of advanced technologies, informed clinical decision-making tools and optimized management protocols with proper training and level of expertise at the ECPR centers. As research evolves, translating these insights into standard clinical practice will be essential for improving patient outcomes and shaping the future landscape of acute cardiac care.
